# Selective suppression of polyglutamine-expanded protein by lipid nanoparticle-delivered siRNA targeting CAG expansions in the mouse CNS

**DOI:** 10.1016/j.omtn.2021.02.007

**Published:** 2021-02-15

**Authors:** Tomoki Hirunagi, Kentaro Sahashi, Kiyoshi Tachikawa, Angel I. Leu, Michelle Nguyen, Rajesh Mukthavaram, Priya P. Karmali, Padmanabh Chivukula, Genki Tohnai, Madoka Iida, Kazunari Onodera, Manabu Ohyama, Yohei Okada, Hideyuki Okano, Masahisa Katsuno

**Affiliations:** 1 Department of Neurology, Nagoya University Graduate School of Medicine, 65 Tsurumai-cho, Syowa-ku, Nagoya, Aichi 466-8550, Japan; 2 Arcturus Therapeutics, 10628 Science Center Drive, Suite 250, San Diego, CA 92121, USA; 3 Department of Neurology, Aichi Medical University School of Medicine, 1-1 Yazakokarimata, Nagakute-shi, Aichi 480-1195, Japan; 4 Department of Dermatology, Keio University School of Medicine, 35 Shinanomachi, Shinjuku-ku, Tokyo 160-8582, Japan; 5 Department of Physiology, Keio University School of Medicine, 35 Shinanomachi, Shinjuku-ku, Tokyo 160-8582, Japan

**Keywords:** siRNA, CAG repeat, selective suppression, polyglutamine diseases, SBMA, androgen receptor, unlocked nucleic acid, lipid nanoparticle

## Abstract

Polyglutamine (polyQ) diseases are inherited neurodegenerative disorders caused by expansion of cytosine-adenine-guanine (CAG)-trinucleotide repeats in causative genes. These diseases include spinal and bulbar muscular atrophy (SBMA), Huntington’s disease, dentatorubral-pallidoluysian atrophy, and spinocerebellar ataxias. Targeting expanded CAG repeats is a common therapeutic approach to polyQ diseases, but concomitant silencing of genes with normal CAG repeats may lead to toxicity. Previous studies have shown that CAG repeat-targeting small interfering RNA duplexes (CAG-siRNAs) have the potential to selectively suppress mutant proteins in *in vitro* cell models of polyQ diseases. However, *in vivo* application of these siRNAs has not yet been investigated. In this study, we demonstrate that an unlocked nucleic acid (UNA)-modified CAG-siRNA shows high selectivity for polyQ-expanded androgen receptor (AR) inhibition in *in vitro* cell models and that lipid nanoparticle (LNP)-mediated delivery of the CAG-siRNA selectively suppresses mutant AR in the central nervous system of an SBMA mouse model. In addition, a subcutaneous injection of the LNP-delivered CAG-siRNA efficiently suppresses mutant AR in the skeletal muscle of the SBMA mouse model. These results support the therapeutic potential of LNP-delivered UNA-modified CAG-siRNAs for selective suppression of mutant proteins in SBMA and other polyQ diseases.

## Graphical Abstract

**Figure:**
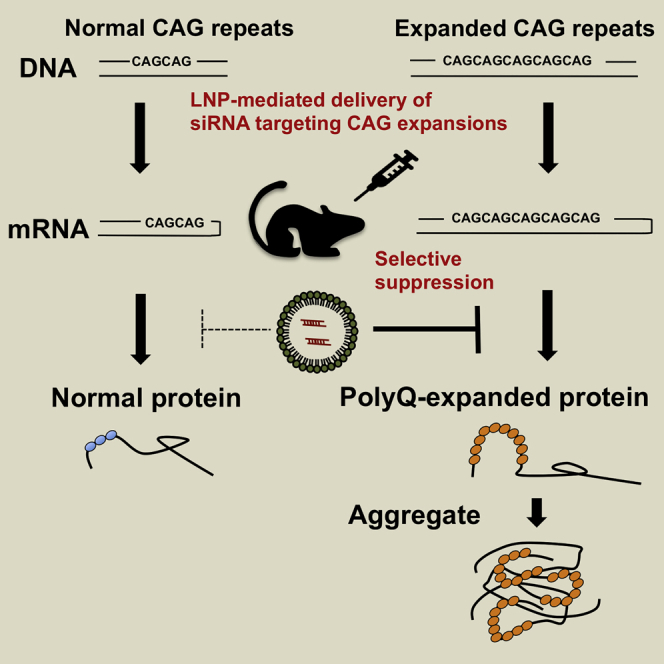

## Teaser

Mutant-allele selective suppression by CAG repeat-targeting oligonucleotides is a promising therapeutic approach to polyglutamine diseases. Hirunagi et al. demonstrate selective suppression of polyglutamine-expanded androgen receptor by lipid nanoparticle-mediated delivery of siRNA targeting CAG expansions in the mouse CNS of an SBMA mouse model.

## Introduction

Spinal and bulbar muscular atrophy (SBMA) is an X-linked and adult-onset neurodegenerative disorder caused by the expansion of trinucleotide cytosine-adenine-guanine (CAG) repeats, which encode a polyglutamine (polyQ) tract in the *androgen receptor* (*AR*) gene.^1^^,^^2^ SBMA belongs to the family of polyQ diseases, which also include Huntington’s disease (HD), dentatorubral-pallidoluysian atrophy (DRPLA), and spinocerebellar ataxia (SCA) types 1, 2, 3, 6, 7, and 17.^3^^,^^4^ The characteristics of polyQ diseases include intracellular aggregates of polyQ-expanded proteins and degeneration of specific neuron populations. For instance, the brainstem and spinal motor neurons in SBMA, striatum and cerebral cortex in HD, and cerebellum in SCA are distinctively affected in the central nervous system (CNS) of patients. Those with polyQ diseases develop progressive neurological symptoms caused by the degeneration of neurons, and currently there is no curative therapy.

Reducing expanded polyQ proteins is a promising therapeutic strategy in polyQ diseases because toxic polyQ proteins play a central role in disease pathogenesis.^3^^,^^4^ The use of antisense oligonucleotides (ASOs) and RNA interference for silencing disease-causing genes has been intensively studied, and clinical trials applying chemically modified ASOs for HD have been launched based on the success of preclinical studies.^5^, ^6^, ^7^ Targeting CAG repeats is another potential therapeutic option in polyQ diseases.^8^ Peptide nucleic acid and locked nucleic acid oligomers complementary to CAG repeats were the first reported oligonucleotides with therapeutic potentials; they showed selective inhibition of expression of the mutant allele of *huntingtin* (*HTT*) and *ataxin* *3* (*ATXN3*).^9^ Further studies showed similar effects produced by ASOs with other types of chemical modification^10^ and small interfering RNA (siRNA) duplexes targeting expanded CAG repeats in HD, SCA3, and DRPLA cell lines.^11^, ^12^, ^13^, ^14^, ^15^ Moreover, recent studies have shown that chemically modified ASOs and single-stranded siRNAs targeting CAG repeats suppress mutant proteins in the CNS of HD,^16^^,^^17^ SCA1, and SCA3^18^ mouse models. However, the knockdown potency and selectivity of CAG repeat-targeting oligonucleotides against mutant AR has yet to be investigated in SBMA.

Given that wild-type proteins with normal glutamine stretches have various functions in the CNS,^19^^,^^20^ selective suppression of mutant proteins with abnormal polyQ expansions is preferable, especially for early therapeutic intervention at presymptomatic stages. In cellular experiments, siRNAs that are fully complementary to CAG repeats showed limited allele selectivity, while those containing central mismatches showed high selectivity for a mutant allele of *HTT*.^12^ ASOs targeting CAG repeats also showed allele selectivity in HD knockin mouse models.^16^^,^^17^ However, the CAG repeats of endogenous mouse *Htt* gene are rather short; thus, it is unclear whether these oligomers can provide allele selectively in HD patients. Another study showed that striatum-injected locked nucleic acid-modified ASOs targeting CAG repeats did not reduce mutant HTT levels in an HD mouse model, although the ASOs ameliorated the phenotype independent of HTT levels.^21^ Overall, selective silencing of mutant proteins by CAG repeat-targeting oligonucleotides is yet to be investigated *in vivo*.

The aim of this study was to explore the potential application of CAG-siRNAs for mutant-allele-selective treatment for polyQ diseases in the context of cellular and mouse models of SBMA. After screening the optimal unlocked nucleic acid (UNA) position using a luciferase-ATXN3 reporter assay, we investigated whether CAG-siRNAs containing a central mismatch and UNA substitutions selectively suppress mutant AR protein in *in vitro* cell models. In addition, we *in vivo* administered a UNA-modified CAG-siRNA into the murine CNS and skeletal muscle using a lipid-enabled and UNA modified RNA (LUNAR) lipid delivery technology platform.

## Results

### Screening the optimal UNA position in CAG-siRNAs with a luciferase-ATXN3 reporter assay

Before testing CAG-siRNAs in cell models of SBMA, we performed a luciferase reporter assay to determine the optimal position of UNA substitutions. We generated luciferase reporter vectors containing human ATXN3 with 24 or 74 expanded CAG repeats (Figure S1A) and screened them to identify siRNA candidates (Figure S1B) by co-transfection of the vectors and each 21-mer CAG-siRNA in HEK293 cells. First, we tested a CAG-siRNA that was fully complementary to the CAG repeats, namely “REP,” and CAG-siRNAs with a central mismatch and different position of a UNA substitution. 5ʹ UNA modifications of the sense strands were used to improve siRNA targeting^22^ in all tested siRNAs (Figure S1B). In accordance with previous results,^14^ the CAG-siRNA with a UNA modification at position 9 from the 5ʹ end of the antisense strand (REPU9) showed high allele selectivity (nanomolar inhibitory concentration 50% (IC_50_) values against wild-type/nanomolar IC_50_ values against mutant [IC_50_ WT/IC_50_ MUT]: 7.84), with a 50% reduction in wild-type ATXN3 expression and 90% reduction in mutant ATXN3 expression at a concentration of 50 nM (Figures S1C and S1D). To further compare the potencies of CAG-siRNAs, we performed a second round of screening using CAG-siRNAs with different UNA modifications, including REP, REPU9, U10, U11, U910, and U1011. Of these, REPU910 showed the highest selectivity (IC_50_ WT/IC_50_ MUT: 46.1), with a 30% reduction in wild-type ATXN3 and 90% reduction in mutant ATXN3 at a concentration of 50 nM (Figures S1E and S1F).

### Selective suppression of mutant androgen receptor by UNA-modified CAG-siRNA in human fibroblasts

**Figure 1:**
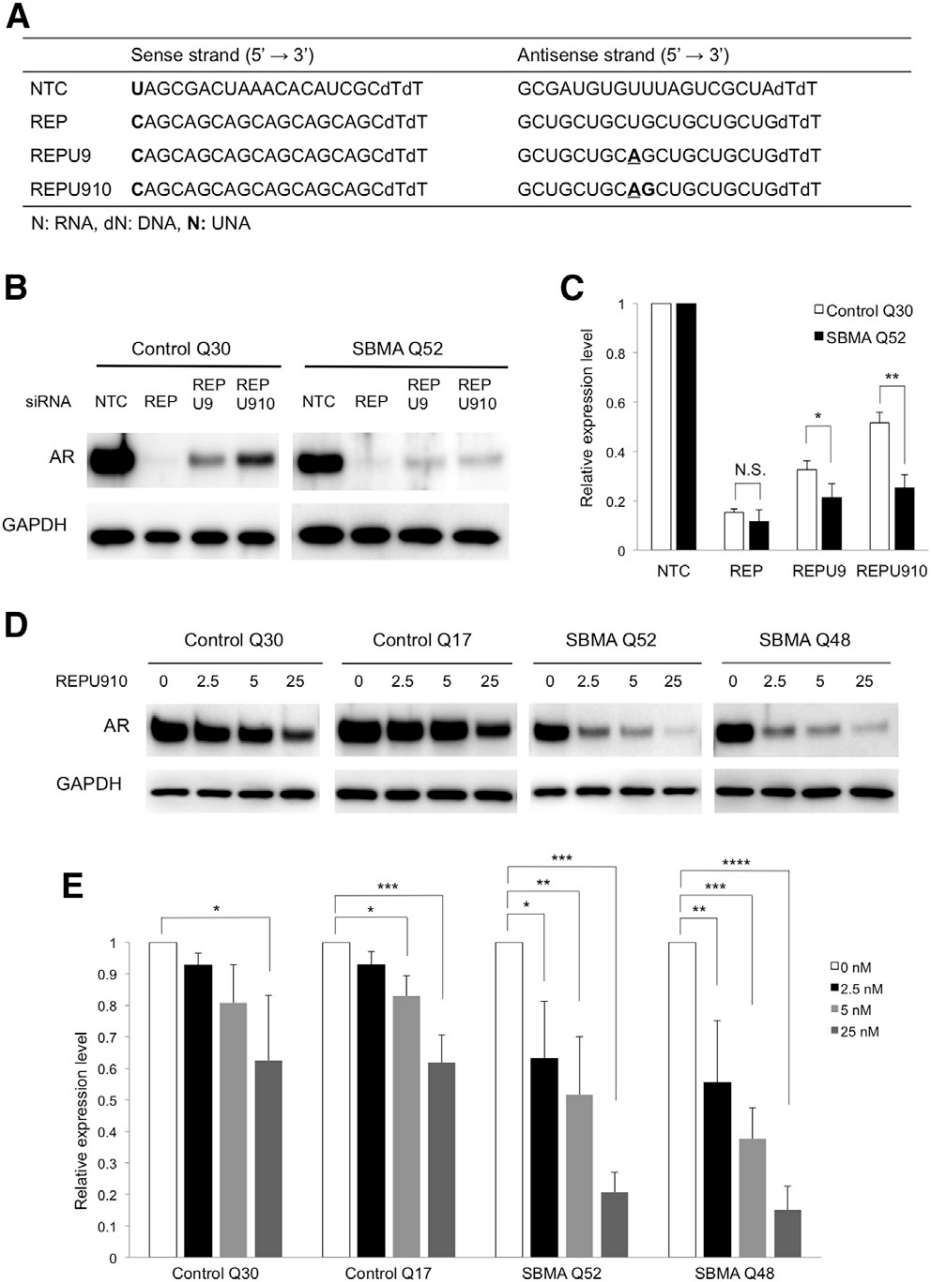
Selective suppression of polyQ-expanded androgen receptor by UNA-modified siRNA targeting CAG repeats (A) The nucleotide sequences and chemical modifications of the siRNA. Unlocked nucleic acid (UNA) substitutions are marked in bold. Underlining represents central mismatches. NTC, negative control. (B) western blot analysis of androgen receptor (AR) levels in healthy control (Q30) and SBMA (Q52) fibroblasts 48 h after transfection with the indicated siRNAs (25 nM). (C) Densitometric quantitation of AR band intensities shown in (B) (n = 3; N.S., not significant; ∗p < 0.05, ∗∗p < 0.01). (D) Knockdown effect of REPU910 siRNA (0, 2.5, 5, and 25 nM) on AR protein levels in healthy control (Q30 and Q17) and SBMA (Q52 and Q48) fibroblasts. (E) Densitometric quantitation of AR band intensities shown in (D) (n = 3 for each concentration; ∗p < 0.05, ∗∗p < 0.01, ∗∗∗p < 0.001, ∗∗∗∗p < 0.0001 One-way ANOVA with post hoc Dunnett’s test). Error bars mean standard deviation.

To clarify whether the UNA-modified CAG-siRNAs also exert allele selectivity for *AR*, we tested REP, REPU9, and REPU910 (Figure 1A) using fibroblasts from an SBMA patient with 52 CAG repeats (SBMA Q52) and a healthy control with 30 CAG repeats (control Q30). *In vitro* siRNA experiments in fibroblasts were performed by lipofection since LUNAR was unsuitable for this *in vitro* use. REP showed the highest knockdown efficiency (>80% reduction) but suppressed both mutant and wild-type AR at a concentration of 25 nM (Figures 1B and 1C). REPU9 and REPU910 suppressed about 65% and 40% wild-type and 80% and 75% mutant AR, respectively (Figures 1B and 1C). Given that REPU910 showed the highest allele selectivity at 25 nM, we also tested it at lower concentrations in fibroblasts derived from two different patients with SBMA (SBMA Q52 and Q48) and two different healthy controls (control Q30 and Q17). Notably, REPU910 had a limited effect on wild-type AR protein expression but suppressed the expression of mutant AR by 40%–65% at concentrations of 2.5 and 5 nM (Figures 1D and 1E). Correspondingly, REPU910 also suppressed *AR* mRNA levels (Figures S2A and S2B). In addition, we tested REPU910 in NSC34 motor neuron-like cells stably expressing wild-type (24Q) and mutant (97Q) *AR*. While showing only a minimal suppression of AR24Q, REPU910 significantly suppressed AR97Q by about 25%, 35%, and 45% at concentrations of 2.5, 5, and 25 nM, respectively (Figures S3A and S3B).

### Detection of intracerebroventricularly (i.c.v.) injected LUNAR-EGFP mRNA

**Figure 2:**
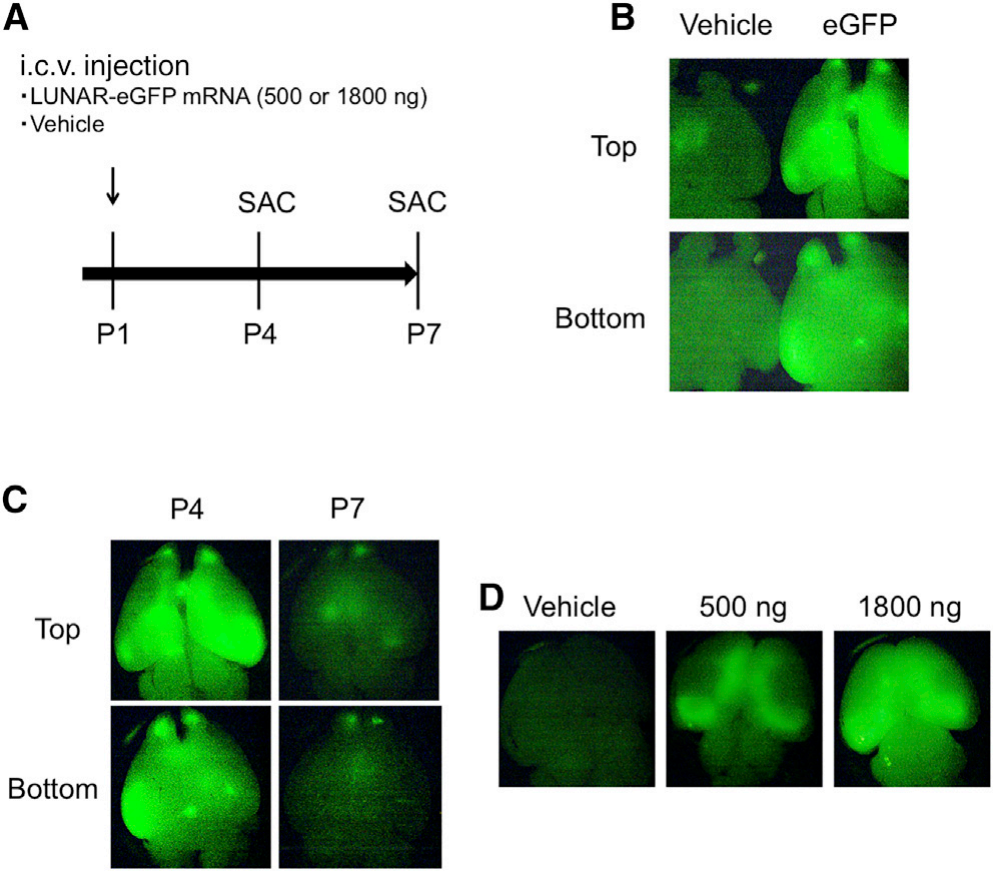
Detection of intracerebroventricularly injected LUNAR-EGFP mRNA (A) Scheme of the experiment. LUNAR-EGFP mRNA (500 or 1,800 ng) complexes or vehicle were intracerebroventricularly (i.c.v.) injected into mice at postnatal day 1 (P1). At P4 or P7, mice were sacrificed, and their brains were dissected. (B) Imaging of EGFP at P4 in whole brains by a fluorescent stereomicroscope. LUNAR-EGFP mRNA (1,800 ng) or vehicle-injected brains were photographed at the same time. (C) Time course of EGFP expression after i.c.v. injection of LUNAR-EGFP mRNA (1,800 ng). (D) Dose-dependent EGFP expression in the brain at P4.

To investigate the applicability of the LUNAR platform in the CNS, we administered LUNAR nanoparticles incorporating 500 or 1,800 ng of EGFP mRNA (denoted LUNAR-EGFP) or vehicle into the lateral ventricle of neonatal mice at postnatal day 1 (P1) and assessed the expression of EGFP fluorescence at P4 or P7 (Figure 2A). At P4, we detected widespread EGFP expression in the brain of LUNAR-EGFP mRNA (1,800 ng)-injected mouse compared to that of vehicle-injected mouse (Figure 2B). At P7, the EGFP expression had decreased but was still detected at low levels (Figure 2C). We observed a weaker signal in the brain of LUNAR-EGFP mRNA (500 ng)-injected mouse compared to that of 1,800 ng-injected mouse (Figure 2D).

### Distribution of LUNAR-EGFP in the CNS

**Figure 3:**
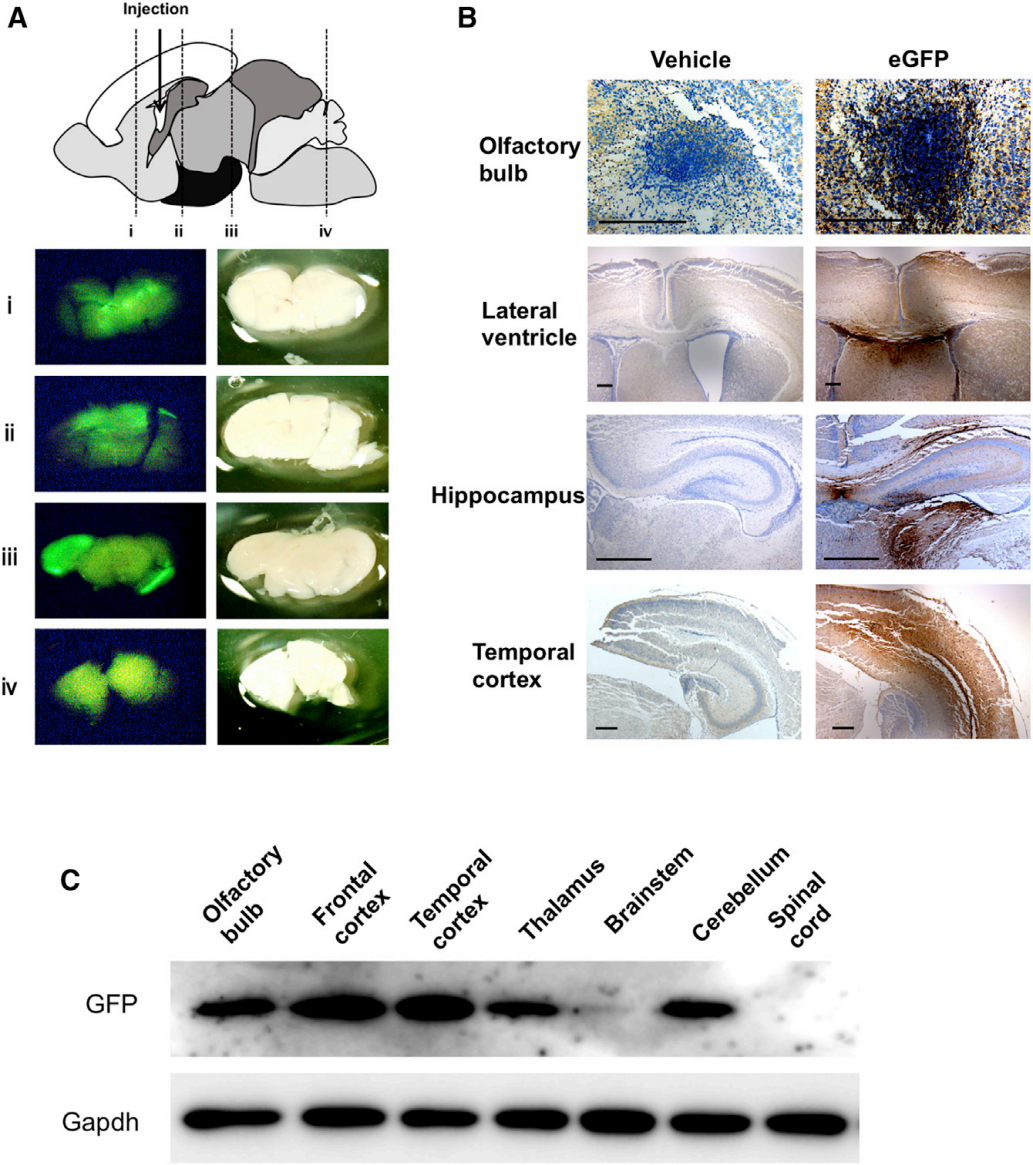
Distribution of LUNAR-EGFP in the CNS (A) Coronal sections of LUNAR-EGFP mRNA (1,800 ng)-injected mouse brains at P4. The upper image is a schematic diagram of the sagittal brain at P4. The lower images are fluorescence imaging of EGFP (left) and stereoscopic imaging (right) in the slices of indicated cut lines (i–iv). (B) The representative images of immunohistochemistry for EGFP in the indicated brain regions. Scale bars, 200 μm. (C) Western blot analysis of EGFP in the indicated brain regions and spinal cord.

We also assessed the detailed distribution of LUNAR nanoparticles in additional experiments. At P4, coronal brain sections of mice that were i.c.v. injected with LUNAR-EGFP at P1 showed strong EGFP signals along the ventricle and in the frontal to temporal cortex as well as relatively weaker signals in other brain regions, such as the thalamus and cerebellum (Figure 3A). Immunohistochemistry for EGFP showed strong immunoreactivity, especially in the olfactory bulb and periventricular area (Figure 3B). Importantly, the finding of EGFP signals in the brain parenchyma confirms that i.c.v.-administered LUNAR nanoparticles penetrate the ependymal cells. Western blotting analysis of EGFP expression showed the following patterns: highest expression in the frontal and temporal cortex; moderate expression in the olfactory bulb, thalamus, and cerebellum; and low or hardly detectable expression in the brainstem and spinal cord (Figure 3C). The expression levels of EGFP in the ipsilateral and contralateral cerebral hemispheres were nearly identical (Figure S4), indicating that i.c.v.-injected LUNAR nanoparticles were evenly distributed across hemispheres.

### LUNAR-EGFP mRNA complexes are incorporated into neurons, astrocytes, and oligodendrocytes

**Figure 4:**
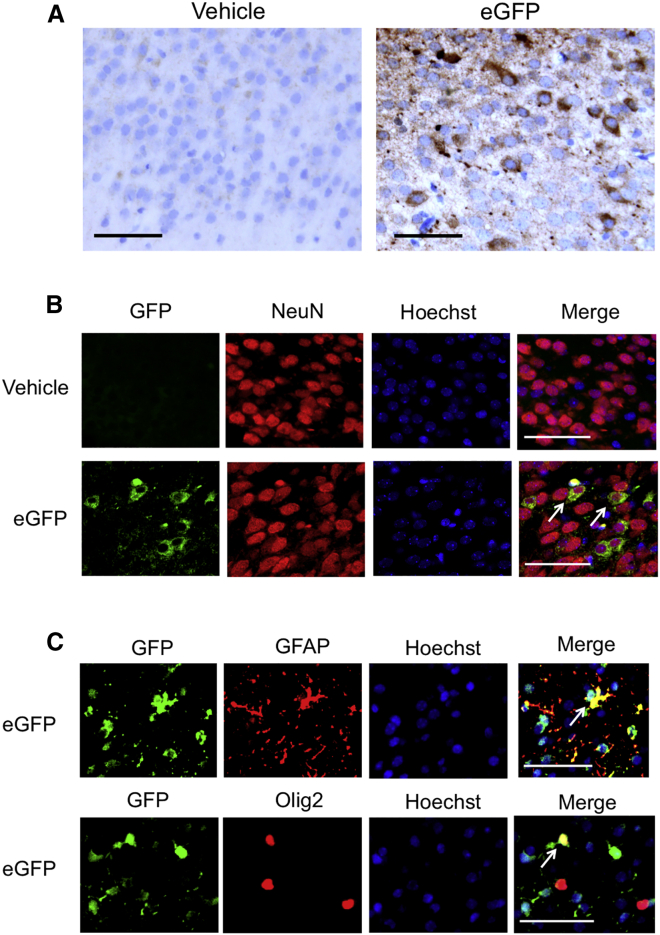
LUNAR-EGFP mRNA complexes are incorporated into neurons, astrocytes, and oligodendrocytes Vehicle or LUNAR-EGFP mRNA (1,800 ng) was i.c.v. injected into mice at P1. (A) Immunohistochemistry in the temporal cortex of mice at P4 shows EGFP expression in the neurons and glial cells. (B and C) Immunofluorescence staining shows EGFP expression in the neurons (B), astrocytes, and oligodendrocytes (C). Hoechst was used for nuclei staining, and NeuN, GFAP, and Olig2 were used as markers for neurons, astrocytes, and oligodendrocytes, respectively. Arrows indicate merged cells. Scale bars, 50 μm.

We further investigated the type of cells that uptake LUNAR nanoparticles. Immunohistochemistry at P4 showed EGFP signals in the neurons and glial cells in the temporal cortex of LUNAR-EGFP-injected mice (Figure 4A). We also performed double immunofluorescence staining for EGFP and cell markers and found that EGFP signals were observed in the neurons, oligodendrocytes, and astrocytes (Figures 4B and 4C). These results suggest not only that LUNAR-EGFP was taken up by both the neurons and glial cells but also that the incorporated EGFP mRNA was efficiently translated by ribosomes.

### Selective suppression of polyQ-expanded AR by i.c.v.-injected LUNAR-REPU910 siRNA

**Figure 5:**
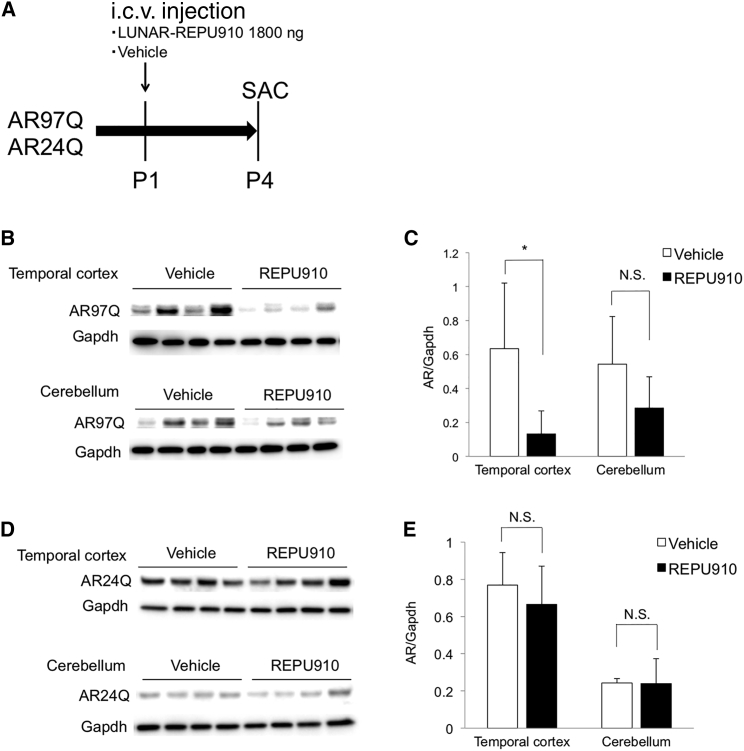
Selective suppression of polyQ-expanded androgen receptor by i.c.v.-injected LUNAR-REPU910 siRNA (A) Scheme of the experiment. LUNAR-EGFP mRNA (1,800 ng) complexes or vehicle were i.c.v. injected into AR24Q or AR97Q mice at P1. At P4, mice were sacrificed, and their brains were dissected. (B) Western blot analysis of AR protein in the temporal cortex and cerebellum of AR97Q mice. (C) Densitometric quantitation of AR97Q band intensities shown in (B) (n = 4; ∗p < 0.05 two-sided t test). (D) Western blot analysis of AR protein in the temporal cortex and cerebellum of AR24Q mice. (E) Densitometric quantitation of AR24Q band intensities shown in (D) (n = 4). N.S., not significant. Error bars mean standard deviation.

Next, we investigated the efficacy of LUNAR-delivered REPU910 siRNA (denoted LUNAR-REPU910) *in vivo*. To assess its mutant allele selectivity, we used our transgenic mice carrying wild-type human *AR* with 24 CAGs (AR24Q mice) and mutant *AR* with 97 CAGs (AR97Q mice).^23^ We i.c.v. injected LUNAR-REPU910 in the mice at P1 and assessed AR protein levels at P4 (Figure 5A). I.c.v. injection of maximum dose (1,800 ng) of REPU910 had no adverse effect on mouse phenotypes, including motor function, growth, and survival. Western blot analysis showed that LUNAR-REPU910 significantly suppressed mutant AR expression by ~90% in the temporal cortex and by ~70%, although not statistically significant, in the cerebellum, compared to vehicle (Figures 5B and 5C). In contrast, no apparent reduction was observed in the temporal cortex and cerebellum of AR24Q mice (Figures 5D and 5E), which demonstrates the allele-selective potency of LUNAR-REPU910 against expanded CAG repeats in *in vivo* mouse brain tissue. Although the precise conversion of the bioavailability of REPU910 is difficult between diverse biological contexts, the pharmacological effects of 1,800 ng of REPU910 siRNA in the temporal cortex and the cerebellum are complementary to those of 5–25 nM and 2.5–5 nM in cell-culture experiments, respectively (Figures 1D, 1E, 5B, and 5C).

### LUNAR-REPU910 suppresses mutant huntingtin in the CNS of R6/2 HD mouse model

To demonstrate the applicability of LUNAR-REPU910 to other polyQ diseases, we used a R6/2 HD mouse model harboring exon 1 *huntingtin* transgene with expanded CAG repeats (MUT-ex1 *HTT*).^24^ PCR analysis^25^ showed that the R6/2 mice used in this study had MUT-ex1 *HTT* containing approximately 160–190 expanded CAGs (Figure S5A). We i.c.v. injected LUNAR-REPU910 (1,800 ng) at P1 and analyzed the MUT-ex1 HTT protein levels at P4. Consistent with previous studies,^26^^,^^27^ western blot analysis revealed that an ~50 kDa band of MUT-ex1 HTT was detected by using 1C2 antibody against expanded polyQ proteins (Figure S5B); LUNAR-REPU910 was found to efficiently suppress MUT-ex1 HTT by ~70% in the temporal cortex of R6/2 mice (Figures S5C and S5D).

### LUNAR-REPU910 suppresses mutant AR in the skeletal muscle in AR97Q mice

**Figure 6:**
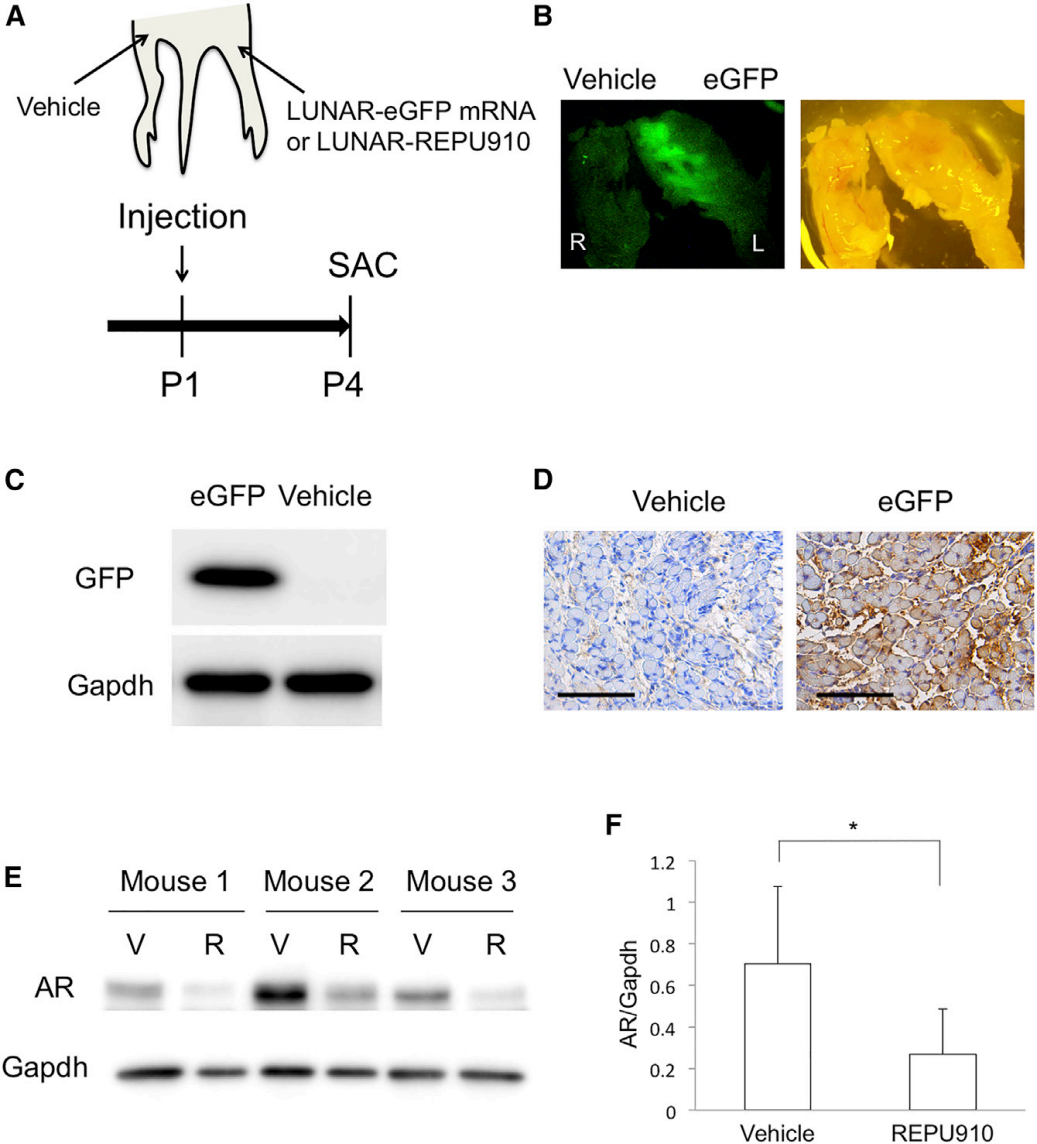
LUNAR-REPU910 siRNA suppresses mutant AR in the skeletal muscle of AR97Q mice (A) Scheme of the experiment. LUNAR-EGFP mRNA (1,800 ng) or LUNAR-REPU910 siRNA (1,800 ng) complexes are subcutaneously injected around the gastrocnemius muscles at P1. Vehicle is injected in the contralateral side. At P4, mice are sacrificed, and their gastrocnemius muscles are dissected. (B) Fluorescence imaging of EGFP (left) and stereoscopic imaging (right) in the hind limbs. LUNAR-EGFP mRNA or vehicle-injected hind limbs were photographed at the same time. (C) Western blot analysis of EGFP protein in the LUNAR-EGFP mRNA or vehicle-injected gastrocnemius muscles. (D) Immunohistochemistry in the gastrocnemius muscles of mice at P4 shows EGFP expression in the muscle cells. Scale bars, 100 μm. (E) Western blot analysis of AR protein in the LUNAR-REPU910 siRNA or vehicle-injected gastrocnemius muscles. V, vehicle; R, LUNAR-REPU910. (F) Densitometric quantitation of AR band intensities shown in (E) (n = 3; ∗p < 0.05 two-sided paired t test). Error bars mean standard deviation.

Finally, we investigated whether LUNAR-REPU910 suppresses mutant AR in the skeletal muscle of AR97Q mice, because peripheral AR suppression is also a candidate approach for the treatment of SBMA.^28^^,^^29^ We subcutaneously injected LUNAR-EGFP mRNA (1,800 ng) into the ipsilateral gastrocnemius muscle at P1 and assessed EGFP expression and distribution at P4. As a control, saline vehicle was simultaneously injected into the contralateral muscle (Figure 6A). Fluorescence imaging of the LUNAR-EGFP-injected hind limb showed high EGFP expression throughout the ipsilateral gastrocnemius muscle but no EGFP signal on the contralateral side (Figure 6B). Western blot analysis confirmed the EGFP expression in the EGFP-treated side of the muscle (Figure 6C). Immunohistochemistry of the muscle showed broad EGFP expression in the myocytes, demonstrating efficient cellular uptake and intracellular distribution of LUNAR nanoparticles (Figure 6D). Similarly, we subcutaneously injected LUNAR-REPU910 (1,800 ng) and saline vehicle to each hind limb at P1 in AR97Q mice and analyzed mutant AR expression levels at P4. Western blot analysis showed that LUNAR-REPU910 obtained a significant (~80%) suppression of mutant AR expression in the gastrocnemius muscle (Figures 6E and 6F).

## Discussion

Using both in *in vitro* cell models and *in vivo* mouse models of SBMA, this study demonstrated the selectivity of UNA-modified CAG-siRNAs for expanded polyQ protein and their potential application in the treatment of polyQ diseases. UNA-modified CAG-siRNAs were shown to achieve selective knockdown of mutant allele expression of *AR* with CAG repeats in human fibroblasts. Consistent with previous reports,^9^^,^^12^ siRNA that was fully complementary to the CAG repeats did not show allele selectivity, whereas mutant-allele selective suppression was obtained by introducing a central mismatch and UNA substitutions in the siRNAs. We uncover that the UNA modifications at positions 9 and 10 (REPU910) achieve especially high selectivity for mutant AR inhibition. UNA is an acyclic analog of RNA with a highly flexible structure.^12^^,^^30^ Both central mismatches and UNA substitutions confer structural flexibility to siRNA duplexes, which would disrupt AGO2-mediated cleavage of target mRNA and provides a mechanism that mimics the action of miRNA in decreasing mRNA stability and translation.^12^^,^^14^^,^^31^ In contrast to a previous study, which reported that UNA-modified CAG-siRNAs caused little alteration of *HTT* mRNA levels in HD patient fibroblasts,^14^ we found that REPU910 siRNA suppressed *AR* mRNA levels, showing a similar tendency of its effect on AR protein levels. The difference between these studies may arise due to diverse effects of CAG-siRNAs on the mRNA of each gene.

We also demonstrated that LNP-delivered REPU910 siRNA selectively suppressed mutant AR expression in the CNS of an SBMA mouse model while preserving the expression of wild-type AR with normal CAG repeats in transgenic mice. To our knowledge, this is the first report of the selective suppression of a polyQ protein by a double-stranded CAG-siRNA *in vivo*. Furthermore, we confirmed that the LNP-delivered REPU910 siRNA also suppressed mutant polyQ-expanded protein in the muscle of the SBMA mouse model as well as in the CNS of HD mouse model. In this study, we used neonatal mice to test the efficacy of LNP-delivered CAG-siRNA, which enables us to assess the *in vivo* potency of CAG-siRNA in an efficient manner, but its therapeutic application to older mice will further provide valuable information for developing treatments for later-onset polyQ diseases.

LNP has attracted great attention as a delivery system for siRNA, and the first LNP-encapsulated siRNA (LNP-siRNA) was recently approved for clinical use in patients with transthyretin amyloidosis.^32^ LNP is accumulated in target cells by apolipoprotein E (APOE)-mediated endocytosis; thus, APOE plays a critical role in the intracellular delivery of siRNAs.^33^ Although the main target tissue of LNP-siRNAs is that of the liver, APOE-mediated uptake of LNP can also occur in the CNS.^34^ Several reports have shown that LNP-siRNA efficiently suppresses target gene expression in the rodent CNS,^35^, ^36^, ^37^ but the potency and detailed distribution of i.c.v.-administered LNP-siRNA had not been fully clarified. In the present study, the widespread distribution of LUNAR nanoparticles was observed across brain regions. Moreover, i.c.v.-injected LUNAR-REPU910 selectively suppressed mutant AR expression in the brain in a glutamine repeat length-dependent manner. As siRNA concentration is not diluted by cell division in non-dividing cells,^38^ the effect of gene suppression can persist for several weeks once siRNA uptake by neurons has occurred.^39^ However, to reduce the frequency of siRNA administration, chemical modifications to increase half-life of oligonucleotides may be useful for clinical applications.

The potential efficacy of anti-androgen therapies against neurological symptoms has been demonstrated in both preclinical and clinical studies in SBMA.^40^ However, the benefit of such therapies is partially limited by accompanying sexual dysfunction and anti-anabolic actions on skeletal muscles; therefore, additional therapeutic strategies are needed. Previous studies have shown that the reduction of mutant AR by systemic or i.c.v.-administered gapmer ASOs ameliorates phenotypes of SBMA mouse models.^28^^,^^41^ Although these ASOs are promising therapeutic candidates, CAG repeat-targeting oligomers, such as REPU910 siRNA, have an advantage in terms of their applicability as common treatments for polyQ diseases. Indeed, we demonstrated that LUNAR-REPU910 enabled efficient suppression of not only polyQ-expanded AR but also polyQ-expanded hungtintin protein in the CNS of mouse models.

A limitation of this study is that i.c.v.-administered LUNAR nanoparticles were barely distributed in the brainstem and spinal cord, so we were unable to assess the potency of REPU910 siRNA in lower motor neurons. In contrast, its potential applicability to the treatment of SBMA was demonstrated by the finding that LUNAR-REPU910 administered subcutaneously enabled suppression of mutant AR expression in the skeletal muscle, another therapeutic target for SBMA.^28^^,^^29^ However, further studies should explore the modification of nanoparticles and siRNAs to obtain the optimal distribution and duration of action to disease tissues for polyQ diseases. We are going to screen our lipid library for optimization of the LNP to enhance the tissue distribution of the siRNA. Otherwise, alternative administration routes, such as intrathecal injection, might improve the pharmacodynamic properties in the CNS.

In conclusion, we showed the selective suppression of mutant AR by UNA-modified CAG-siRNA in *in vitro* SBMA cell models and demonstrated that LNP-mediated delivery of the CAG-siRNA selectively reduces mutant AR levels in the *in vivo* brain of an SBMA mouse model. Furthermore, the LNP-delivered CAG-siRNA also suppressed mutant huntingtin in the CNS and mutant AR in the skeletal muscle in mice. These results provide a proof of concept of LNP-delivered CAG-siRNA for selective suppression of expanded polyQ protein in SBMA, as well as its broad applicability to other polyQ diseases.

## Material and methods

### Preparation of siRNA and mRNA

All the siRNA sequences used in this study were synthesized by Integrated DNA Technologies (Coralville, IA, USA). Co-transcriptionally capped EGFP mRNA was synthesized by T7 RNA polymerase using HiScribe T7 High Yield RNA Synthesis Kit from New England Biolabs (Ipswich, MA, USA) according to manufacturer’s instructions. Purity and concentration of the mRNA were measured by 2100 Bioanalyzer (Agilent Technologies, Santa Clara, CA, USA) and NanoDrop Spectrophotometer (ThemoFisher Scientific, Waltham, MA, USA), respectively.

### Preparation of LUNAR-mRNA and LUNAR-siRNA nanoparticles

LUNAR nanoparticles encapsulating REPU910 siRNA or EGFP mRNA were prepared by mixing an ethanolic solution of lipids with an aqueous solution of RNA as previously described.^42^ Briefly, lipid excipients (ATX-Arcturus Therapeutics proprietary ionizable lipid, DSPC, cholesterol and PEG2000-DMG) are dissolved in ethanol at mole ratio of 58:7:33.5:1.5 (for siRNA) and 50:7:40:3 (for mRNA). An aqueous solution of the RNA is prepared in citrate buffer pH 3.5. The lipid mixture is then combined with the RNA solution at a flow rate ratio of 1:3 (v/v) using the NanoAssemblr microfluidic system (Precision NanoSystems). Nanoparticles thus formed are dialyzed against either Tris or HEPES buffer pH 7.4 using a 100,000 molecular weight cut-off (MWCO) dialysis tube (Repligen) at room temperature. Concentration of the formulation is adjusted to the final target RNA concentration using 100,000 MWCO Amicon Ultra centrifuge tubes (Millipore Sigma) followed by filtration through a 0.2 μm polyethersulfone sterilizing-grade filter. Post filtration, bulk formulation is aseptically filled into sterile Eppendorf tubes and frozen at −70 ± 10°C. Analytical characterization includes measurement of particle size and polydispersity using dynamic light scattering (ZEN3600, Malvern Instruments) and RNA content and encapsulation efficiency by a fluorometric assay using Ribogreen RNA reagent (Thermo Fisher Scientific).

### Construction of luciferase reporter vectors

Open reading frames for human ATXN3 with either 24 or 74 CAG repeats were PCR-amplified using genomic DNA isolated from Machado-Joseph disease patient’s fibroblasts with ATXN3-specific primers containing either the Pml I or RsrG I site; they were then cloned in-frame downstream of firefly luciferase in the psiVer3 vector, which was constructed based on the psiCHECKTM2 vector (Promega, Madison, WI, USA), as shown in Figure S1. The resulting pArc vectors also contained a constitutively expressed *Renilla* luciferase gene, which served as an internal control to normalize transfection efficiency.

### Luciferase reporter assay

The Dual-Luciferase Reporter Assay System (DLR assay system, Promega, Madison, WI, USA) was used to perform dual-reporter assays on psiCHECK2-based reporter systems. A total of 5,000 HEK293 cells (American Type Culture Collection) were plated onto a well of 96-well plate 1 day before the transfection. The cells were incubated at 37°C in 100 μL of DMEM (Life Technologies, Carlsbad, CA, USA) supplemented with 0.1 mM nonessential amino acids and 10% FBS (Life Technologies, Carlsbad, CA, USA). The reporter plasmid and siRNA were co-transfected with transfection reagent: Lipofectamine 3000 (Life Technologies, Carlsbad, CA, USA) was used to transfect the reporter plasmid (25 ng), and various amounts of siRNA together with P3000 were transfected into the cells according to the manufacturer’s protocol. One day after transfection, the cells were gently washed once with phosphate-buffered saline. Subsequently, 40 μL per well of Passive Lysis Buffer (Promega, Madison, WI, USA) was added to the cells, which were then incubated with gentle rocking for 20 min at room temperature. Luciferase activities were measured using a Cytation 3 imaging reader (BioTek, Winooski, VT, USA), and the effects of the siRNA on reporter expression were calculated based on the ratio of firefly/*Renilla*, which was used to normalize cell number and transfection efficiency.

### Cell culture and transfection

Dermal fibroblasts were collected at biopsy from genetically confirmed SBMA patients. Healthy control human fibroblasts were obtained from Kurabo Industries. The length of CAG repeats was determined by fragment analysis and Sanger sequencing of the PCR products.^43^ NSC34 motor neuron-like cells stably expressing AR24Q or AR97Q were established as previously described.^44^ Cells were maintained in DMEM supplemented with 10% FBS. Fibroblasts and NSC34 cells were plated in 6-well and 24-well plates 24 h before transfection, respectively. SiRNAs were transfected with Lipofectamine RNAiMax transfection reagent (Invitrogen, Carlsbad, CA, USA) according to the manufacturer’s instructions. Transfected cells were then cultured in DMEM with 10% FBS and 50 nM dihydrotestosterone. RNA and protein were isolated 48 h after transfection.

### Animals

Transgenic mice bearing chicken β-actin promoter-driven human AR with elongated or normal length of CAG repeat (AR97Q and AR24Q) were generated and maintained on a C57BL/6J background in the animal facility.^23^^,^^44^ The mice, genotyped using PCR of tail DNA, were allowed free access to water and a standard diet and maintained on a 12 h light/dark cycle.^23^ We mated AR97Q or AR24Q mice to wild-type C57BL/6J mice and randomly allocated newborn mice of them to each treatment group. Transgenic mice bearing human *HTT* promoter-driven human MUT-ex1 *HTT* (R6/2) were generated and maintained on a CBA × C57BL/6J background.^24^ We obtained R6/2 mice from the Jackson Laboratory, and their embryos were frozen and stored until use. We generated newborn mice by frozen embryo transfer into recipient Institute of Cancer Research mice and randomly allocated them to each treatment group. R6/2 mice were genotyped using PCR of tail DNA and the following primers for determining the presence of MUT-ex1 HTT transgene: forward/reverse, 5ʹ-CCGCTCAGGTTCTGCTTTTA-3ʹ/5ʹ-TGGAAGGACTTGAGGGACTC-3ʹ, and the size of the CAG repeat lengths in the transgene: forward/reverse 5ʹ- CCGCTCAGGTTCTGCTTTTA-3ʹ/5ʹ-GGCTGAGGAAGCTGAGGAG-3ʹ. We used approximately 40 neonatal mice in total throughout this study. The number of mice used in each experiment is described in each figure legend.

### i.c.v. injection

The procedure of i.c.v. injection was previously described.^45^ Briefly, P1 neonatal mice were cryoanesthetized on ice, and 2 μL of LUNAR-mRNA or LUNAR-siRNA in saline containing Fast Green FCF (0.01% [w/v]; Sigma-Aldrich, St. Louis, MO, USA) was injected into the left lateral ventricle using a 5 μL microsyringe (Hamilton Company, Reno, NV, USA) and a 33-gauge needle.

### Subcutaneous injection

We subcutaneously injected LUNAR-mRNA or LUNAR-siRNA in saline containing Fast Green FCF (0.01% [w/v]; Sigma-Aldrich) around the gastrocnemius muscles of the left hind limb using a 5-μL microsyringe (Hamilton Company) and a 33-gauge needle. Vehicle was similarly injected in the same site of the right hind limb.

### Protein isolation and western blotting

After mice were sacrificed, their brain regions and spinal cord were dissected and snap-frozen in powdered CO_2_ in acetone. Protein was isolated from mouse tissue and fibroblasts using Cellytic MT Cell Lysis Reagent (Sigma-Aldrich) supplemented with Halt Protease and Phosphatase Inhibitor Cocktails (Thermo Scientific, Waltham, MA, USA). We separated equal amounts of protein on 5%–20% SDS-PAGE gels (Wako, Osaka, Japan) and transferred them to Hybond-P membranes (GE Healthcare, Piscataway, NJ, USA). The following antibodies were used in this study: anti-AR (1:2,000; H280, Santa Cruz Biotechnology, Santa Cruz, CA, USA), anti-GAPDH (1:5,000; 6C5, Abcam, Cambridge, MA, USA), anti-GFP (1:1,000; D5.1, Cell Signaling Technology, Beverly, MA, USA), and anti-polyQ 1C2 (1:2,000; MAB1574, Millipore, Billerica, MA, USA). The density of each band was quantified using ImageJ software (NIH, Bethesda, MD, USA).

### Immunohistochemistry and immunofluorescence

After mouse brains were dissected, they were immediately fixed in a 10% buffered formalin solution. Sections (3 μm) were then deparaffinized, heated in a microwave for 15 min in a 10 mM citrate buffer (pH 6.0), and incubated overnight with the following primary antibodies: anti-GFP (1:200; D5.1, Cell Signaling Technology), anti-NeuN (1:100; MAB377, Millipore), anti-GFAP (1:1,000; ab53554, Abcam), and anti-Olig2 (1:250; MABN50, Millipore). For immunohistochemistry, the samples were incubated with a secondary antibody labeled with a polymer as part of the Envision+ system containing horseradish peroxidase (Dako Cytomation, Glostrup, Denmark). Photographs of immunohistochemically stained sections were captured using an optical microscope (BX51, Olympus). For immunofluorescence, the samples were incubated for 1 h with the following secondary antibodies: Alexa 488-conjugated goat anti-rabbit IgG (1:1,000; Invitrogen), Alexa-546-conjugated goat anti-mouse IgG (1:1,000; Invitrogen), and biotinylated horse anti-goat IgG (1:500; BA-9500, Vector Laboratories). Biotinylated secondary antibodies were visualized using Alexa-594-conjugated streptavidin (1:1,000; Invitrogen). Nuclei were stained with Hoechst 33342 (1:400; Invitrogen). Immunofluorescence-stained sections were mounted with ProLong Gold (Invitrogen) and then imaged with a fluorescent microscope (BZ-X710, Keyence, Osaka, Japan).

### RNA isolation and PCR

Total RNA was extracted from the fibroblasts using TRIzol (Invitrogen) and RNeasy Mini Kit (QIAGEN, Hilden, Germany). The extracted RNA was then reverse-transcribed into first-strand cDNA using the ReverTra Ace qPCR RT Kit (Toyobo, Osaka, Japan). PCR amplification was performed using rTaq DNA polymerase (Toyobo) according to the manufacturer’s protocol. Quantitative PCR was performed using THUNDERBIRD SYBR qPCR Mix (Toyobo), and the amplified products were detected with the iCycler system (Bio-Rad Laboratories, Hercules, CA, USA). To analyze AR and GAPDH transcripts, the following PCR primers were used: AR forward/reverse, 5ʹ-CGGAAGCTGAAGAAACTTGG-3ʹ/5ʹ-ATGGCTTCCAGGACATTCAG-3ʹ; GAPDH forward/reverse 5ʹ-GAGTCAACGGATTTGGTCGT-3ʹ/5ʹ-TTGATTTTGGAGGGATCTCG-3ʹ.

### Statistical analysis

We analyzed the data with an unpaired two-sided t test for comparisons of two groups and the one-way ANOVA with post hoc Dunnett’s test for multiple comparisons unless otherwise noted. Analyses were conducted in GraphPad Prism8 (GraphPad, San Diego, CA, USA).

### Study approval

The collection of biopsied human fibroblasts and their use in this study was approved by the ethics committees of the Nagoya University Graduate School of Medicine (approval no. 2019-0406), the Keio University School of Medicine (approval no. 20080016), and the Aichi Medical University School of Medicine (approval no. 14-004). All animal experiments were performed in accordance with the National Institute of Health Guide for the Care and Use of Laboratory Animals and with the approval of the Nagoya University Animal Experiment Committee (no. 20049).

## Supplemental information

Document S1. Figures S1–S5

Document S2. Article plus supplemental information
