# Supplementary material for: Selective suppression of polyglutamine-expanded protein by lipid nanoparticle-delivered siRNA targeting CAG expansions in the mouse CNS
Source: Mol Ther Nucleic Acids. 2021 Feb 15;24:1–10. doi: 10.1016/j.omtn.2021.02.007 (PMC7937577; doi:10.1016/j.omtn.2021.02.007)
Supplement: Document S1. Figures S1–S5 [file mmc1.pdf]

OMTN, Volume *24*

Supplemental information

Selective suppression of polyglutamine-expanded protein by lipid nanoparticle-delivered siRNA targeting CAG expansions in the mouse CNS

Tomoki Hirunagi, Kentaro Sahashi, Kiyoshi Tachikawa, Angel I. Leu, Michelle Nguyen, Rajesh Mukthavaram, Priya P. Karmali, Padmanabh Chivukula, Genki Tohnai, Madoka Iida, Kazunari Onodera, Manabu Ohyama, Yohei Okada, Hideyuki Okano, and Masahisa Katsuno

A

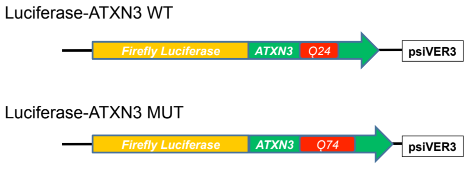

B

	Sense strand (5' → 3')
	**C**AGCAGCAGCAGCAGCAGCdTdT
	Antisense strand (5' → 3')
REP	GCUGCUGCUGCUGCUGCUGdTdT
REPU3	GC**U**GCUGCAGCUGCUGCUGdTdT
REPU5	GCUG**C**UGCAGCUGCUGCUGdTdT
REPU7	GCUGCU**G**CAGCUGCUGCUGdTdT
REPU9	GCUGCUGC**A**GCUGCUGCUGdTdT
REPU10	GCUGCUGCA**G**CUGCUGCUGdTdT
REPU11	GCUGCUGCAG**C**UGCUGCUGdTdT
REPU13	GCUGCUGCAGCU**G**CUGCUGdTdT
REPU15	GCUGCUGCAGCUGC**U**GCUGdTdT
REPU910	GCUGCUGC**AG**CUGCUGCUGdTdT
REPU1011	GCUGCUGCA**GC**UGCUGCUGdTdT

N: RNA, dN: DNA, **N:** UNA

C

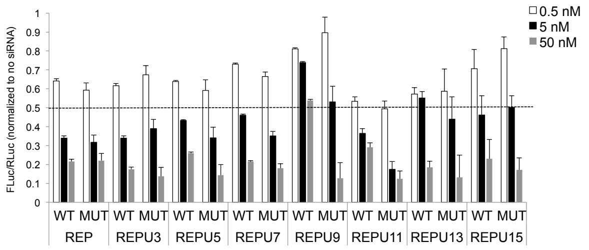

D

1st round screening

	IC50 WT/IC50 MUT
REP	1.38
REPU3	0.65
REPU5	2.25
REPU7	2.06
**REPU9**	**7.84**
REPU11	1.98
REPU13	2.09
REPU15	0.77

E

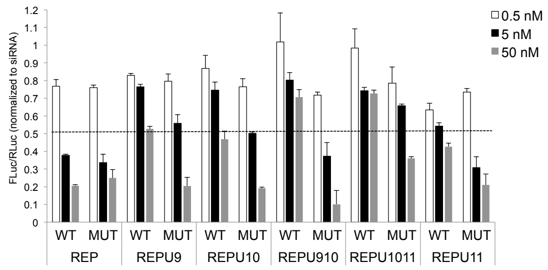

F

2nd round screening

	IC50 WT/IC50 MUT
REP	1.16
REPU9	6.98
REPU10	7.18
**REPU910**	**46.1**
REPU1011	7.24
REPU11	3.97

Figure S1. Screening optimal UNA position in CAG-siRNA using luciferase-ataxin 3 reporter assay

(A) Scheme of luciferase-ataxin 3 (ATXN3) vectors. Wild-type (WT) and mutant (MUT) ATXN3 contain 24 and 74 CAG repeats, respectively. (B) The nucleotide sequences and chemical modifications of tested siRNAs. UNA substitutions are marked in bold. Underlining represents a central mismatch. (C) The normalized ratio of firefly to Renilla luciferase activity (FLuc/RLuc) in the first screening luciferase-ATXN3 reporter assay in HEK293 cells. (D) Selectivity (IC50 WT/IC50 MUT) of first round screening of siRNAs. (E) The normalized FLuc/RLuc in the second screening luciferase-ATXN3 reporter assay in HEK293 cells. (F) Selectivity (IC50 WT/IC50 MUT) of second round screening of siRNAs.

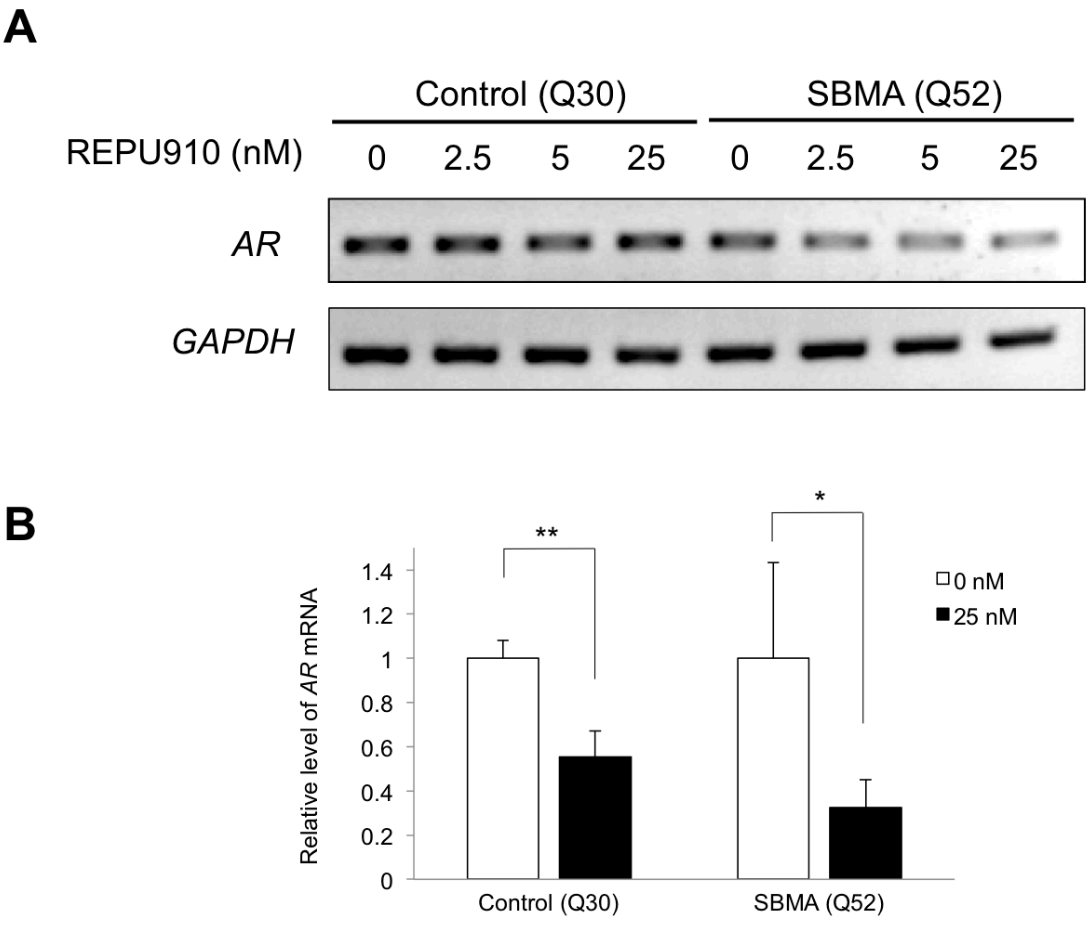

Figure S2. Knockdown effect of REPU910 siRNA on AR mRNA levels in healthy control Q30 and SBMA Q52 fibroblasts

(A) REPU910 siRNA suppresses AR mRNA levels and shows a similar tendency of its effect on protein levels (shown in Figure 1D). (B) Quantitative PCR analysis of AR mRNA levels after transfection with 0 or 25 nM REPU910 siRNA (n = 3, * $p < 0.05$, **$p < 0.01$).

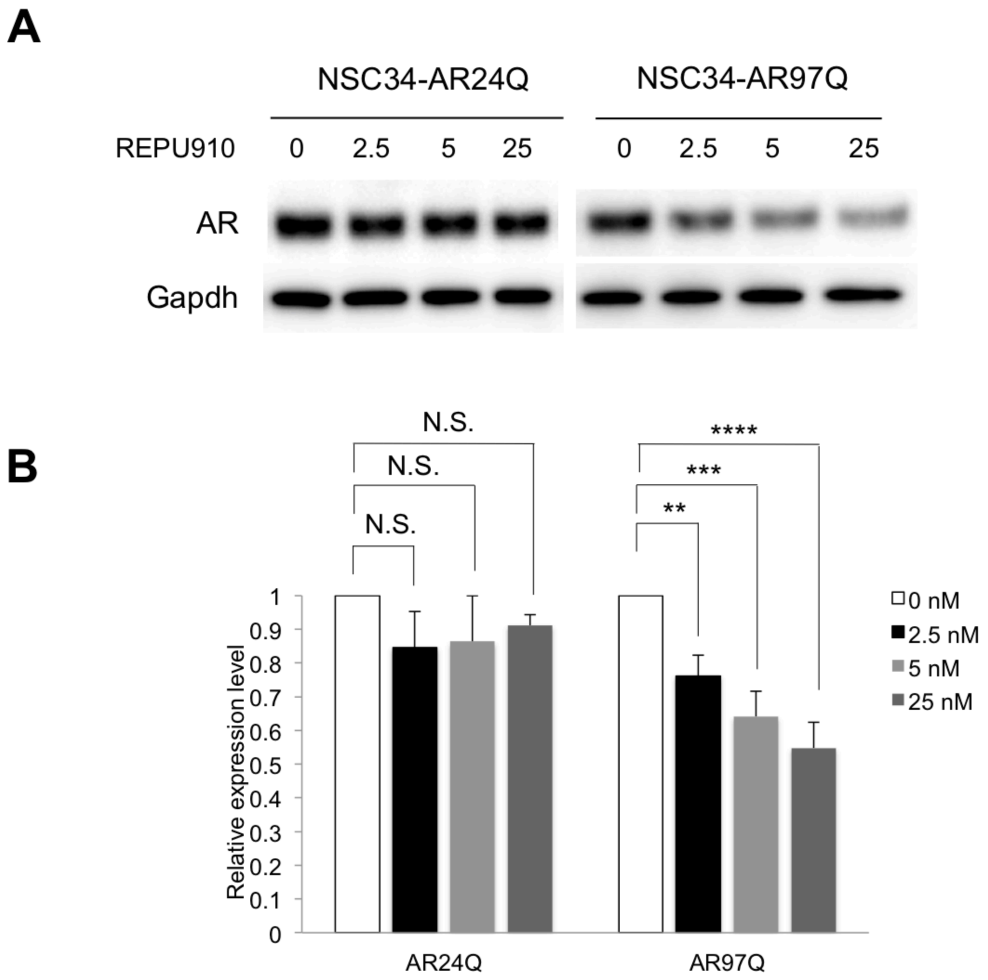

Figure S3. Selective suppression of polyglutamine-expanded androgen receptor by REPU910 siRNA in NSC34 motor neuron-like cells

(A) Knockdown effect of REPU910 siRNA (0, 2.5, 5, and 25 nM) on AR protein levels in NSC34 expressing wild-type (24Q) and mutant (97Q) AR. (B) Densitometric quantitation of AR band intensities shown in (A) ($n = 3$ for each concentration, N.S.: not significant, $^{**}p < 0.01$, $^{***}p < 0.001$, $^{****}p < 0.0001$ One-way ANOVA with post-hoc Dunnett's test).

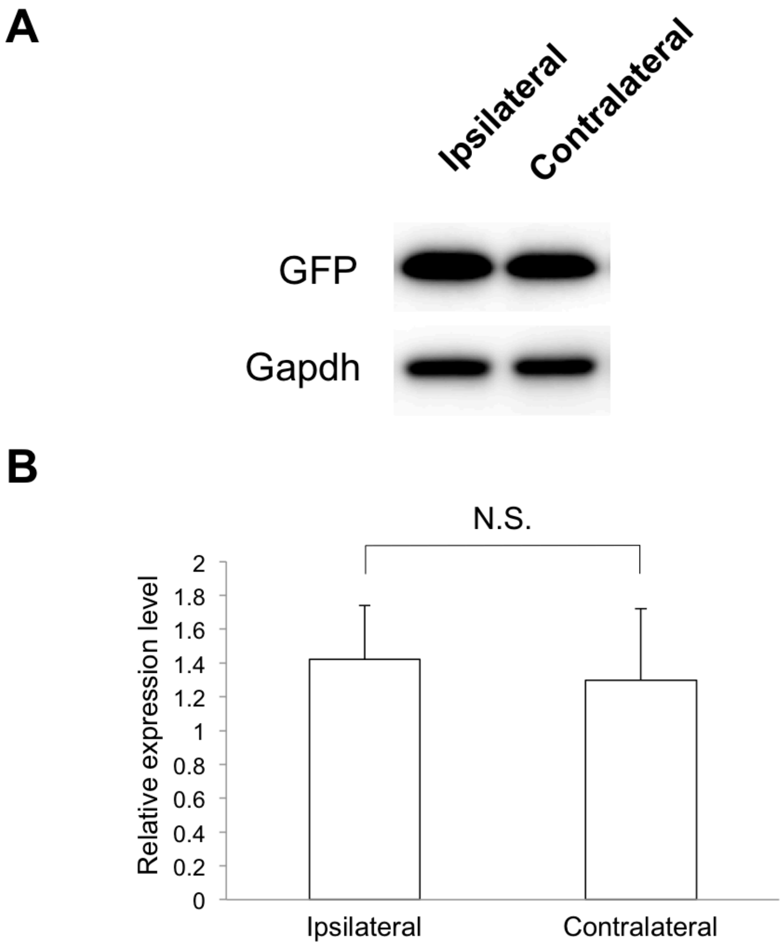

Figure S4. Expression levels of eGFP in the ipsilateral and contralateral cerebral hemispheres of LUNAR-eGFP-injected mice

(A) Western blot analysis of eGFP protein in the ipsilateral and contralateral cerebral hemispheres of LUNAR-eGFP (1800 ng)-injected mice. (B) Densitometric quantitation of eGFP band intensities shown in (A) (n = 4, N.S.: not significant, two-sided paired t test).

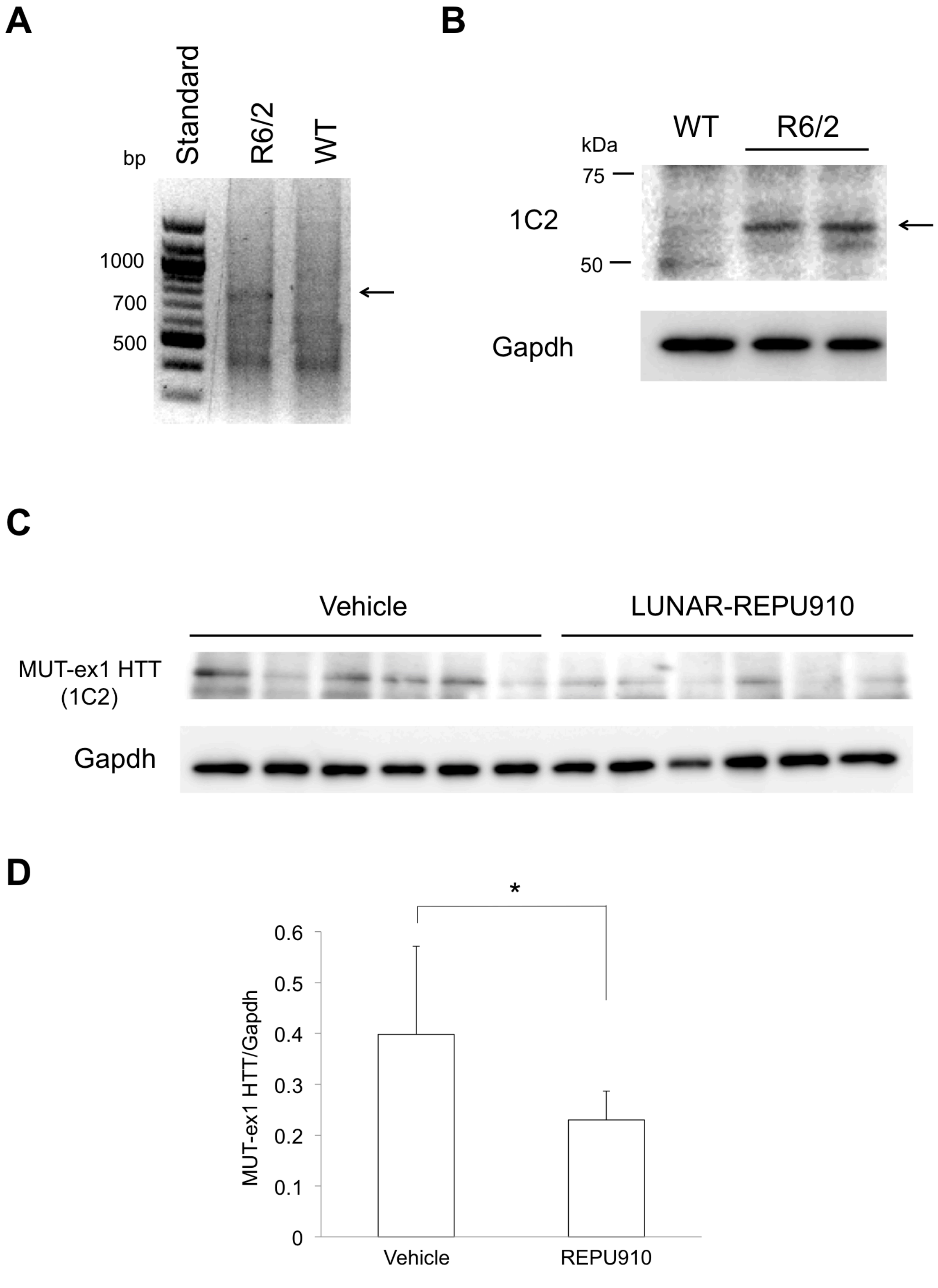

Figure S5. LUNAR-REPU910 suppressed mutant exon 1 huntingtin in the CNS of R6/2 HD model mice

(A) PCR analysis of tail genomic DNA for the determination of the CAG repeat lengths shows a band between 700 and 800 bp (arrow), indicating that R6/2 mice used in this study harbor mutant exon 1 huntingtin (MUT-ex1 *HTT*) with approximately 160–190 CAG repeats. (B) Detection of MUT-ex1 HTT protein by Western blot analysis using 1C2 antibody (arrow). (C) Western blot analysis of MUT-ex1 HTT protein in the temporal cortex of LUNAR-REPU910 (1800 ng)-injected R6/2 mice. (D) Densitometric quantitation of MUT-ex1 HTT band intensities shown in (C) (n = 6, * $p < 0.05$).
